# Renal biopsy cases in myeloproliferative neoplasms (MPN)

**DOI:** 10.1007/s13730-013-0067-0

**Published:** 2013-03-12

**Authors:** Kumi Fujita, Kazuhiro Hatta

**Affiliations:** 1 Tenri Institute of Medical Research, 200 Mishima-cho, Tenri, Nara Japan; 2 Tenri Hospital, Department of General Medicine, 200 Mishima-cho, Tenri, Nara Japan

**Keywords:** Polycythemia vera, Essential thrombocytosis, Renal biopsy, Glomerulonephritis, Antihypertensive agent

## Abstract

We performed renal biopsy in three cases complicated by myeloproliferative neoplasms (MPN). Although several cases of glomerulonephritis associated with MPN have been reported, the etiologies of the renal disorders were not established (Plomley et al., Aust NZ J Med, 13:125–129, 1983; Sharma et al., Nephron, 69:361, 1995; Kanauchi et al., Intern Med, 33:36–40, 1994; Kasuno et al., Nephrol Dial Transplant, 12:212–215, 1997; Au et al., Am J Kid Dis, 34:889–893, 1999; Kosch et al., Nephrol Dial Transplant, 15:1710–1711, 2000; Oymak et al., Nephron, 86:346–347, 2000; Chun et al., Am J Nephrol, 20:344–346, 2000; Chung et al., Am J Nephrol, 22:397–401, 2002; Asaba et al., Clin Exp Nephrol, 7:296–300, 2003; Haraguchi et al., Clin Exp Nephrol, 10:74–77, 2006; Saigusa et al., J Nephrol, 19:656–658, 2006; Okuyama et al., Clin Nephrol, 6:412–415, 2007; Nishi et al., Clin Nephrol, 5:393–398, 2010; Ulusoy et al., Intern Med, 49:2477, 2010). A review of previous reports of renal biopsy cases with MPN in the English literature suggested that circulation control is important for the treatment of renal disorders that mimic glomerulonephritis in MPN.

## Introduction

Although several cases of glomerulonephritis with myeloproliferative neoplams (MPN) such as polycythemia vera (PV) or essential thrombocythemia (ET) have been reported, the association between MPN and glomerulonephritis is still obscure. The purpose of this article was to evaluate whether MPN can cause glomerulonephritis.

We describe three MPN cases in which renal biopsies were performed. Paraffin sections from the biopsied specimens of the three cases were examined with hematoxylin and eosin, periodic acid-Schiff, methenamine silver, and Masson’s trichrome stains. Immunoglobulins (IgG, IgA, and IgM), complement (C3 and C1q), and fibrinogen were assessed by a direct immunofluorescence staining method on frozen sections for two cases and by immunostaining on paraffin sections for the other case. Electron microscopy was performed for all three cases.

Here, we also review previously reported cases in the English literature of renal glomerular disorders associated with MPN, which were examined by renal biopsy.

## Case reports

### Case 1

A 76-year-old woman with a history of bronchial asthma complained of edema. She had been diagnosed with thrombocytosis due to ET 3 years earlier. Her condition was stable at diagnosis, but edema occurred 2 months later, upon which administration of hydroxyurea was initiated.

The patient’s height, weight, and blood pressure were 150 cm, 50 kg, and 180/80 mmHg, respectively. She was alert and appeared healthy. She did not have any remarkable heart, lung, or abdominal abnormalities. Pitting edema was demonstrated in her lower extremity.

The laboratory data were as follows: hemoglobin, 15.8 g/dl; hematocrit, 47.3 %; platelets, 490,000/μl; white blood cells, 8,100/μl, C-reactive protein, <0.2 mg/dl; blood urea nitrogen, 25.7 mg/dl; serum creatinine, 1.2 mg/dl; total cholesterol, 182 mg/dl; total protein, 5.9 g/dl; albumin, 3.1 g/dl; globulin, 2.8 g/dl; lactate dehydrogenase, 241 IU/l; aspartate aminotransferase, 18 IU/l; alanine aminotransferase, 12 IU/l; total bilirubin, 0.3 mg/dl; γ-glutamyl transpeptidase, 25 IU/l; alkaline phosphatase, 284 IU/l; Na, 141 mmol/l; K, 4.8 mmol/l; and Cl, 109 mmol/l. Urinalysis showed 3+ proteinuria and 2+ hematuria.

**Fig. 1:**
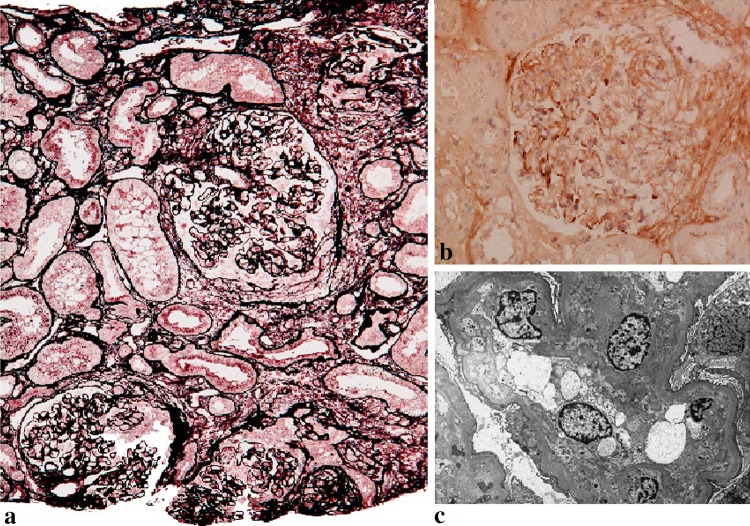
Case 1. **a** Micrograph of the glomeruli showing mesangial proliferation and crescent formations. **b** Immunoglobulin A deposition in the mesangial region of a glomerulus identified by immunostaining. **c** Electron-dense deposits in the mesangial region

Renal biopsy was performed (Fig. 1); 13 glomeruli could be evaluated, 2 of which exhibited global sclerosis. Moderate mesangial proliferation with several crescents and arteriolosclerotic changes were observed. Immunohistological staining was positive for IgG and IgA with a granular pattern and negative for IgM, C1q, C3, and fibrinogen. Electron microscopy showed electron-dense deposits in the mesangium. The patient was diagnosed with IgA nephropathy with an active lesion, and treatment consisting of 30 mg prednisolone, 50 mg cyclophosphamide, and 80 mg telmisartan per day was started. Although treatment improved the proteinuria and hematuria, the patient’s serum creatinine level remained unchanged. Telmisartan decreased her blood pressure, but her hematocrit level could not be normalized. The patient experienced a complicated acute myocardial infarction 3 years later. She died 5 years later from ischemic bowel disorder and heart failure.

### Case 2

A 62-year-old woman was admitted to our hospital because of general fatigue and chest discomfort. She had experienced acute glomerulonephritis 6 years previously, but the details of the clinical course were not known. Proteinuria had been detected while she was pregnant, and she had been treated for hypertension for 15 years. She had been diagnosed with ET at another hospital 3 years earlier. No remarkable cardiac problems were found when she was examined at the Department of Cardiology after admission. She was referred to the Department of General Medicine for a renal biopsy to investigate the persistent proteinuria.

The patient’s height, weight, and blood pressure were 148 cm, 55 kg, and 179/107 mmHg, respectively. Her consciousness level was alert and she looked well. A systolic murmur was detected at the 4 left sternal border. She did not have remarkable abnormalities in the lungs and abdomen and did not show pitting edema.

The laboratory data were as follows: hemoglobin, 11.4 g/dl; hematocrit, 39.8 %; platelets, 978,000/μl; white blood cells, 14,400/μl; C-reactive protein, <0.2 mg/dl; blood urea nitrogen, 15.8 mg/dl; serum creatinine, 0.7 mg/dl; total cholesterol, 227 mg/dl; total protein, 7.3 g/dl; albumin, 4.5 g/dl; globulin, 2.8 g/dl; lactate dehydrogenase, 811 IU/l; aspartate aminotransferase, 22 IU/l; alanine aminotransferase, 11 IU/l; total bilirubin, 0.3 mg/dl; γ-glutamyl transpeptidase, 21 IU/l; alkaline phosphatase, 470 IU/l; Na, 144 mmol/l; K, 5.0 mmol/l; and Cl, 107 mmol/l. Urinalysis showed 4+ proteinuria (1.51 g/day) and no evidence of hematuria.

**Fig. 2:**
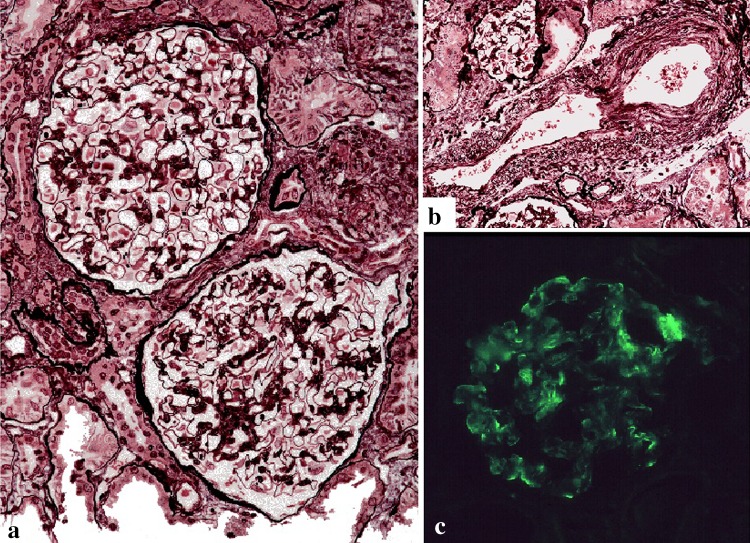
Case 2. **a** Micrograph of the glomeruli showing mesangial proliferation. **b** Interlobular artery with intimal thickening. **c** Immunoglobulin M deposition in the mesangial region of a glomerulus identified by immunostaining

Renal biopsy was performed (Fig. 2), and 19 glomeruli could be evaluated, 2 of which exhibited global sclerosis. The histological findings included mild glomerular changes with arteriolosclerotic changes. Immunofluorescence staining was positive for IgM and weakly positive for IgA. Electron microscopy did not show any obvious electron-dense deposits. She was treated with felodipine, an antihypertensive, as well as antiplatelet drugs. The control of her blood pressure was slightly poor. Her renal function remained stable, but the proteinuria persisted.

### Case 3

A 51-year-old man was admitted to our hospital for renal biopsy. Proteinuria had been detected when the patient was approximately 30 years of age, and he had experienced tonsillitis several times. The patient had been diagnosed with PV with an abnormality of the *JAK2* gene 3 years earlier. Renal biopsy was planned because his proteinuria had become more apparent over the past 2 years.

The patient’s height, weight, and blood pressure were 165 cm, 66 kg, and 120/73 mmHg, respectively. His consciousness level was alert, and he appeared healthy. No remarkable abnormalities were found in the heart, lungs, or abdomen, and pitting edema was not observed.

The laboratory data were as follows: hemoglobin, 15.3 g/dl; hematocrit, 48.1 %; platelets, 326,000/μl; white blood cells, 8,400/μl; C-reactive protein, <0.2 mg/dl; blood urea nitrogen, 22.2 mg/dl; serum creatinine, 1.4 mg/dl; total cholesterol, 194 mg/dl; total protein, 7.1 g/dl; albumin, 4.1 g/dl; globulin, 3.0 g/dl; lactate dehydrogenase, 169 IU/l; aspartate aminotransferase, 17 IU/l; alanine aminotransferase, 23 IU/l; total bilirubin, 0.5 mg/dl; γ-glutamyl transpeptidase, 21 IU/l; alkaline phosphatase, 276 IU/l; Na, 139 mmol/l; K, 4.7 mmol/l; and Cl, 106 mmol/l. Urinalysis showed 4+ proteinuria (1.2 g/day) and no evidence of hematuria.

**Fig. 3:**
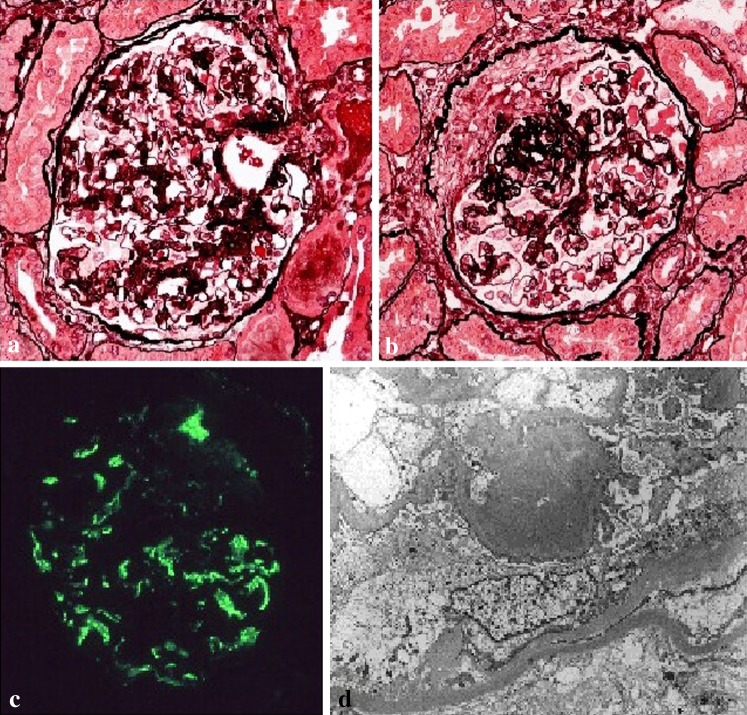
Case 3. **a** Micrograph of the glomeruli showing mesangial proliferation. **b** Micrograph of the glomeruli showing a fibrocellular crescent formation. **c** Immunoglobulin A deposition in the mesangial region of a glomerulus identified by immunostaining. **d** Electron-dense deposits in the paramesangial region

Renal biopsy was performed (Fig. 3) and 22 glomeruli could be evaluated, 5 of which showed global sclerosis. Histological findings included mesangial proliferative glomerulonephritis with crescents and focal segmental necrosis. Arteriosclerotic changes were also observed. Immunofluorescence staining was positive and revealed strong immunoreactivity for IgA and C3. Electron microscopy showed electron-dense deposits in the mesangium. He was diagnosed with IgA nephropathy. Tonsillectomy plus steroid pulse therapy and an angiotensin II receptor blocker were selected as treatments for the patient. His proteinuria and hematuria disappeared after 6 months.

## Discussion

According to the World Health Organization classification, MPNs are classified into the following six diseases: PV, ET, chronic myelogenous leukemia (CML), primary myelofibrosis (PMF), chronic neutrophilic leukemia (CNL), and chronic eosinophilic leukemia/hypereosinophilic syndrome (CEL/HES) [16]. Most reports of glomerular disease associated with MPN have involved PV or ET, whereas few reports have described associations with PMF, CML, CNL, or CEL/HES. We limited our subjects to cases with PV or ET in this study and found 21 appropriate cases in the English literature between 1980 and 2010 [1–15].

The 21 cases in the literature and our 3 cases are shown in Table 1 [1–15].

**Table 1:** Cases of renal glomerular disease associated with MPN in the English literature

	References	Age	Sex	Type	Pathological diagnosis	Pathological findings	Hb (g/dl)	Plt (/μl)	WBC (/μl)	sCr (mg/dl)	Proteinurea (g/day)	Blood pressure (mmHg)	Therapy	Response to therapy	Time course^b^
1	Plomley [1]	47	M	PV		Mes	22.0	310000	8000		2.3	150/110	Antihypertensive agent, phlebotomy	Improved	
2	Plomley [1]	50	M	PV		Mes	18.8	1300000	12000		Not documented	165/110	Antihypertensive agent, phlebotomy, BUS	Worsened	
3	Plomley [1]	45	F	PV		Mes	22.0	215000	8000		1.1	170/120	Antihypertensive agent, phlebotomy, BUS	No change	
4	Kanauchi [3]	53	M	PV	HSP	Mes Cre	8.8	1550000	48800		Not documented	150/90	Phlebotomy, BUS, MCNU, PSL, mPSL (pulse), CPA	Worsened	2 years
5	Sharma [2]	40	F	PV	FSGS	Mes	20.0	410000^a^	32000^a^		2.0	160/100	Phlebotomy	Improved	3 years
6	Kasuno [4]	35	M	PV	IgAN	Mes	21.6	421000	15400	1.6	1.6	224/140	Antihypertensive agent, phlebotomy, PSL, mPSL(pulse)	Improved	5 years
7	Kasuno [4]	51	M	PV	IgAN	Mes	20.6	1155000	17800	1.0	1.0	Not documented	Not documented	Not documented	4 years
8	Au [5]	48	M	PV	FSGS	Segmental sclerosis	21.3	714000	12000		2.5	Hypertension (no data)	Phlebotomy, HU	Worsened	20 years
9	Au [5]	63	F	PV	FSGS	Segmental sclerosis	18.7	1068000	11400		5.4	Hypertension (no data)	Phlebotomy, HU	Worsened	
10	Au [5]	25	M	ET	FSGS	Mes	18.0	118000	27800		2.2	Hypertension (no data)	HU, anti-platelet agent	No change	
11	Au [5]	39	F	ET	FSGS	Segmental sclerosis	10.6	954000	10400		5.0	Hypertension (no data)	HU	No change	
12	Kosch [6]	52	M	PV	FSGS	Tip lesion	18.2	410000	10300	1.1	4.0	160/90	Phlebotomy	Improved	4 years
13	Oymak [7]	66	M	PV		Mes Epi Endo Cre	20.8	660000	17900	7.5	Not documented	160/100	Phlebotomy, PSL, CPA	Improved	
14	Chun [8]	58	M	PV	MN	MN	21.8	210000	9610	0.8	4.4	190/110	Antihypertensive agent (ACEI), PSL, CPA	Improved	
15	Chung [9]	46	M	PV	IgAN	Mes Cre	21.7	258000	19000	2.7	9.1	180/110	Antihypertensive agent (ACEI), HU	Improved	
16	Asaba [10]	68	M	ET	FGN	FGN		1220000		1.8	7.5	Normal (no data)	Antihypertensive agent (ARB), PSL, mPSL (pulse)	No change	28 years
17	Haraguchi [11]	76	M	ET	FSGS	Foamy cell infiltration	14.6	871000	25510	1.0	12.7	Not documented	No treatment	Not documented	
18	Saigusa [12]	75	M	ET	FSGS	Segmental sclerosis	12.7	1040000	9800	1.0	9.2	146/68	HU	Improved	5 years
19	Okuyama [13]	69	F	PV	FSGS	Segmental sclerosis	18.4	689000	14300	1.0	8.3	180/88	Antihypertensive agent, HU, PSL	Improved	3 years
20	Nishi [14]	72	F	PV	MPGN	Mes	11.6	781000	15780	0.5	6.1	140/80	Antihypertensive agent (ARB), phlebotomy, HU, anti-platelet agent	Improved	13 years
21	Ulusoy [15]	56	M	PV	FSGS	Segmental sclerosis	15.2	879000	11500	1.9	6.0	160/90	Antihypertensive agent (ACEI), phlebotomy	Improved	
22	Case 1	76	F	ET	IgAN	Mes Cre	15.8	490000	8100	1.2	No data	180/90	Antihypertensive agent (ARB), PSL, CPA	Improved	2 years
23	Case 2	62	F	ET		Mes	11.4	978000	14400	0.7	1.5	179/107	Antihypertensive agent, anti-platelet agent	No change	
24	Case 3	51	M	PV	IgAN	Mes Cre	15.3	326000	8400	1.4	1.2	120/73	Antihypertensive agent (ARB), tonsillectomy, PSL	Improved	

*Mes* mesangial proliferation, *Cre* crescents formation, *IgAN* IgA nephropathy, *FSGS* focal segmenta glomerulosclerosis, *MN* membranous glomerulonephritis, *FGN* fibrally glomerulonephritis, *MPGN* membranous proliferative glomerulonephritis, *ACEI* angiotensin-converting enzyme inhibitor, *ARB* angiotensin II receptor blocker, *BUS* busulfan, *MCNU* ranimustine, *HU* hydroxycarbamide, *PSL* prednisolone, *mPSL* methylprednisolone, *CPA* cyclophosphamide

^a^In units of “/cm,” it is the text itself

^b^Time course from onset of MPD to renal biopsy. In 13 cases, renal biopsies were performed when MPDs were diagnosed almost simultaneously, so spaces are not occupied

The average patient age of these 24 cases (16 men and 8 women) was 55.1 years. PV was found in 17 patients and ET in 7 patients. Of the 24 cases, 5 cases, including 2 of ours, were diagnosed with IgA nephropathy (IgAN) after renal biopsy: focal segmental glomerulosclerosis (FSGS) in 10 cases, membranous glomerulonephritis in 1 case, membranoproliferative glomerulonephritis in 1 case, purpura nephritis in 1 case, and fibrillary glomerulonephritis in 1 case. The other 5 cases were not specified. Hypertension was observed in 20 of 24 cases. Treatments consisted of antihypertensive drugs for 13 patients, phlebotomy for 12 patients, corticosteroid for 8 patients, hydroxycarbamide for 8 patients, and cyclophosphamide for 4 patients. Urinalysis improvement was seen in 13 cases, 9 of which were treated with antihypertensive agents, 7 with phlebotomy, and 6 with corticosteroid. Four cases worsened despite treatment.

Cases of glomerular disease associated with MPN have been reported occasionally. Although Au et al. [5] detected 5 cases of FSGS (3.6 %) among 138 patients with MPN, the frequency of glomerular disorders among MPN patients is unknown. Therefore, reports that various types of glomerular disease are associated with MPN indicate that the pathogenesis of this condition is complex. Au et al. suggested that high platelet counts and abnormal activation of megakaryocytes are predisposing factors for glomerulosclerosis and that aggressive treatment to lower the platelet count can prevent the development of renal abnormalities [5]. On the other hand, antihypertensive agents, such as an angiotensin II receptor blocker (ARB), were selected for treatment because of complication of hypertension. Ulusoy reported the successful treatment of 1 case with antihypertensive drug administration and phlebotomy [15]. A good response after phlebotomy was observed in 2 cases [3, 6]. MPN, especially PV and ET, are susceptible to complication by thrombosis or hypertension [17]. Alternation of renal hemodynamics in patients with MPN might cause glomerular endothelial injury. Endothelial cell damage could induce activation of platelets and release of cytokines such as PDGF (platelet-derived growth factor). Floege et al. [18] suggested that PDGF might mediate glomerular injury and cause exacerbation of glomerulonephritis. They clarified that PDGF could induce mesangial and endothelial cell proliferation. Other candidate cytokines such as insulin-like growth factor-1, platelet-activating factor, transforming growth factor-β, and plasmalemmal vesicle-associated protein-1 had been reported to play a role in the pathogenesis of glomerulonephritis in MPN patients, though a definite role for these factors has not been established [2, 4, 5, 14]. Sharma et al. [2] and Kosch et al. [6] reported that improvement of hematocrit levels might be useful for reducing protein urea and recovering renal function. Controlling renal hemodynamics and blood cell proliferation might be important for improving renal disease in MPN patients. Unfortunately, with regard to our cases, the effectiveness of these treatments could not be clarified.

Regarding the clinical course, glomerulonephritis was thought to have preceded MPN in 2 of our cases (cases 2 and 3). In case 1, a case of IgA nephropathy, the onset of IgA nephropathy did not occur until 2 years after the PV diagnosis. Although the documented onset of MPN preceded renal disease in 9 of the 21 previously reported cases, a period of 10 years or more passed between the onset of MPN and that of renal disease in 3 cases, indicating that an association between MPN and the onset of renal disease is unlikely. In those cases, glomerular diseases were considered to simply overlap with MPN. However, MPN might exacerbate glomerular disease because of the alternation of renal hemodynamics at least in part.

## References

[1] Plomley RF, Kincaid-Smith PS, Sullivan JR, Fairley KF, Whitworth JA, Brown RW (1983). Polycythemia vera and glomerulonephritis. Aust NZ J Med.

[2] Sharma RK (1995). Focal segmental glomerulosclerosis in a patient with polycythemia rubra vera. Nephron.

[3] Kanauchi M, Dohi K, Shiiki H, Fujii Y, Ishikawa H (1994). Henoch-Shönlein purpura nephritis associated with polycythemia vera. Intern Med.

[4] Kasuno K (1997). IgA nephropathy associated with polycythemia vera: accelerated course. Nephrol Dial Transplant.

[5] Au WY, Chan KW, Lui SL, Lam CCK, Kwong YL (1999). Focal segmental glomerulosclerosis and mesangial sclerosis associated with myeloproliferative neoplasms. Am J Kid Dis.

[6] Kosch M (2000). Focal sclerosis with tip lesions secondary to polycythaemia vera. Nephrol Dial Transplant.

[7] Oymak O (2000). Polycythemia vera presenting with rapidly progressive glomerulonephritis and pyoderma gangrenosum. Nephron.

[8] Chun SL (2000). Secondary polycythemia associated with idiopathic membranous nephropathy. Am J Nephrol.

[9] Chung J, Park PG, Song K (2002). IgA nephropathy in a patient with polycythemia vera. Am J Nephrol.

[10] Asaba K (2003). Fibrillary glomerulonephritis associated with essential thrombocytosis. Clin Exp Nephrol.

[11] Haraguchi K (2006). Focal segmental glomerulosclerosis associated with essential thrombocythemia. Clin Exp Nephrol.

[12] Saigusa T (2006). A case of essential thrombocytosis developing nephrotic syndrome and severe endothelial damage. J Nephrol.

[13] Okuyama S (2007). Focal segmental glomerulosclerosis associated with polycythemia vera: report of a case and review of the literature. Clin Nephrol.

[14] Nishi Y (2010). Histopathological manifestations of membranoproliferative glomerulonephritis and glomerular expression of plasmalemmal vesicle-associated protein-1 in a patient with polycythemia vera. Clin Nephrol.

[15] Ulusoy S (2010). Absence of hypoalbuminemia despite nephrotic proteinuria in focal segmental glomerulosclerosis secondary to polycythemia vera. Intern Med.

[16] Swerdlow S, Campo E, Lee Harris N, Jaffe E, Pileri S, Stein H, et al. WHO classification of tumors of hematopoietic and lymphoid tissues, 4th ed. Lyon: IARC Press; 2008.

[17] Landolfi R, Di Gennaro L (2012). Thrombosis in myeloproliferative and myelodysplastic syndrome. Hematology.

[18] Floege J (1992). Glomerular cell proliferation and PDGF expression precede glomerulosclerosis in the remnant kidney model. Kidney Int.

